# The *CTLA4 *variants may interact with the *IL23R*- and *NOD2*-conferred risk in development of Crohn's disease

**DOI:** 10.1186/1471-2350-11-91

**Published:** 2010-06-10

**Authors:** Ondrej Hradsky, Petra Dusatkova, Martin Lenicek, Jiri Bronsky, Jiri Nevoral, Libor Vitek, Milan Lukas, Ivana Zeniskova, Ondrej Cinek

**Affiliations:** 1 Department of Pediatrics, University Hospital Motol and Second Faculty of Medicine, Charles University in Prague, Prague, Czech Republic; 2 Institute of Clinical Biochemistry and Laboratory Diagnostics, First Faculty of Medicine, Charles University in Prague, Prague, Czech Republic; 3 IBD Clinical and Research Center, ISCARE I.V.F. Lighthouse, Prague, Czech Republic; 4 České Budějovice Hospital a.s., České Budějovice, Czech Republic

## Abstract

**Background:**

The *CTLA4 *(cytotoxic T-lymphocyte antigen 4) gene is associated with several immunopathologic diseases and because of its important immuno-regulatory role it may be considered also a plausible candidate for a genetic association with inflammatory bowel diseases. Previously published studies found no association of *CTLA4 *with Crohn's disease itself, but some indicated an association with its subphenotypes. The aim of this study was to assess the association in the Czech population, using a set of markers shown to associate with other diseases.

**Methods:**

Six polymorphisms within the *CTLA4 *region were investigated in 333 patients with Crohn's disease and 482 unrelated healthy controls, all Caucasians of Czech origin. The genotypes of the SNPs were determined using the TaqMan SNP genotyping assays. Haplotypes were reconstructed using an expectation-maximization algorithm, and their association with the condition was assessed using log-linear modeling. Then, potential interactions were tested between the *CTLA4 *variants and other genetic factors known to confer the disease susceptibility.

**Results:**

No crude associations with Crohn's disease were found for the tested *CTLA4 *variants under the log-additive or dominant models. However, when stratified for the genetic risk conferred by the variants in the *NOD2 *(the p.Leu1007fsX1008, rs5743293) or the *IL23R *(p.R381Q, rs11209026), a significant negative association emerged for the minor alleles of *CTLA4 *CT60 (rs3087243), JO31 (rs11571302), JO27-1 (rs11571297) polymorphisms. This negative association with *CTLA4 *was apparent only in the strata defined by presence minor alleles at the *NOD2 *rs5743293 (here the *CTLA4 *CT60 A coffered an OR = 0.43, 95%CI 0.19 - 0.95 for the presence of CT60 A), or *IL23R *rs11209026 (here the OR for presence of CT60 A was 0.23, 95%CI 0.07 - 0.71). We observed this effect also for the haplotype consisting of minor alleles of the three tightly linked *CTLA4 *markers. Furthermore, this haplotype was associated with the younger age at diagnosis (OR 1.52, 95%CI 1.09 - 2.11, p = 0.014).

**Conclusions:**

A protective effect of a *CTLA4 *haplotype was unmasked after stratification for the risk variants in the *NOD2 *and *IL23R *genes, and may point towards the biological relevance of the molecule in the pathogenesis of the disease.

## Background

Crohn's disease (CD) belongs to inflammatory bowel diseases (IBD) that are characterized by chronic, relapsing and recurrent inflammation of intestinal mucosa. The disease is thought to result from the action of environmental factors in genetically susceptible individuals. Three variants in the *NOD2 *[1,2], IBD5 locus [3] and one variant in the *IL23R *[4] and in the *ATG16L1 *[5] have been independently confirmed to be associated with CD, including associations found previously in the Czech population [6,7]. Recent studies, however, show that this list is far from being complete [5,8-12].

The *CTLA4 *gene may also be considered as a plausible candidate for a genetic association with IBD. Its product, the cytotoxic T-lymphocyte-associated protein 4 (CTLA4) is a T-cell suppressor which plays an essential role in the function of the CD25(+)CD4(+) regulatory cells that control the process of intestinal inflammation [13,14]. The *CTLA4 *gene maps within the 2q33 region that has been found to carry suggestive linkage significance for IBD [15]. The *CTLA4 *gene is associated with other immunopathologic diseases (type 1 diabetes, Graves' disease, Addison's disease, celiac disease, systemic lupus erythematosus, rheumatoid arthritis, vitiligo) [16]. Among the studied single nucleotide polymorphisms (SNPs), the CT60 (rs3087243) shows the most prominent associations, being followed by other three SNPs: JO31 (rs11571302), JO30 (rs7565213) and JO27-1 (rs11571297) [16]. A recent publication has shown evidence for association of another SNP within the *CTLA4 *with type 1 diabetes, the rs1427676 [17]. Previously published papers about genetic association with CD tested three variants in the *CTLA4 *gene: g.49A > G (rs231775), g.-318C > T (rs5742909) and the previously mentioned CT60, having found no association [18-21]. However, several works suggested that *CTLA4 *variants may influence the phenotype of CD [18,19].

The aim of this study was to assess the association in the Czech population, using a set of markers previously shown to associate with other diseases.

## Methods

### Subjects

In a case-control design, 333 Czech patients were compared to 482 unrelated healthy Czech controls representing a general population sample from the same geographical region. We tested 137 pediatric-onset patients (71 boys, 66 girls) who developed CD under or at the age of 18 years and were diagnosed according to the Porto criteria [22], and 196 adult onset patients (77 males, 119 females) diagnosed according to endoscopic, radiological, histological and clinical criteria. Phenotypic classification was done according to the Montreal Classification [23]. The demographic and clinical characteristics of the patients are listed in Table 1 and Table 2. The control group included 482 individuals: 295 children, 187 adult; 311 males, 171 females; median age 12 years, interquartile range 7-34 years. The study was approved by the Ethics Committees of the authors' institutions, and a written informed consent was obtained from all participants or their guardians.

**Table 1:** Demographic characteristics of patients and control subjects

	CD patients			Control subjects (n = 482)
	Total (n = 333)	Pediatric-onset CD (n = 137)	Adult-onset CD (n = 196)	
Sex, M/F	148/185	71/66	77/119	311/171
Age, median (interquartile range)	21 (14-30)1	14 (12-16)1	28 (23-35)1	12 (7-34)2

1: Age at diagnosis

2: Age at enrolment

**Table 2:** Clinical characteristics of patients

	Total (n = 333)	Pediatric-onset CD (n = 137)	Adult-onset CD (n = 196)
**Age at diagnosis**			
A1 (<17 years)	111	111	0
A2 (17 - 40 years)	190	26	164
A3 (>40 years)	32	0	32
**Localization**			
L1 (terminal ileum)	71 (21%)	23 (17%)	48 (25%)
L2 (colon)	50 (15%)	13 (9.5%)	37 (19%)
L3 (ileocolon)	208 (63%)	101 (74%)	107 (55%)
L 1-3 not determined	4	0	4
L4 (Upper GI)^1^	56 (17%)	20 (15%)	36 (18%)
**Disease behavior**			
B1 (nonstricturing/nonpenetraiting)	138 (42%)	79 (59%)	59 (30%)
B2 (stricturing)	129 (39%)	34 (25%)	95 (49%)
B3 (penetrating)	62 (18%)	21 (16%)	41 (21%)
B 1-3 not determined	4	3	1
B4 (perianal disease)	109 (33%)	32 (23%)	77 (39%)
Extraintestinal manifestation^2^	53 (16%)	21 (15%)	32 (17%)
Need for surgery^3^	173 (52%)	41 (30%)	132 (68%)

1: GI: gastrointestinal.

2: Either of: peripheral arthritis, ankylosing spondylitis, sacroilitis, episcleritis and iritis, erythema nodosum, pyoderma gangrenosum, or sclerosing cholangitis.

3: Abdominal surgery for complication of CD (resection)

### Genotyping

Genomic DNA was extracted from peripheral blood leukocytes with a routine salting out method, or from salivary samples using Oragene DNA Self-Collection Kit according the manufacturer's protocol (DNA Genotek Inc., Ottawa, Ontario, Canada). One SNP proximal to *CTLA4 *(rs736611), one from within the gene (g.49G > A, rs231775), and four SNPs located distally from the coding part of *CTLA4 *gene (rs3087243 also called CT60; rs11571302 called JO31; rs11571297 called JO27-1; and rs1427676) were selected based on available literature and genotyped using the TaqMan SNP genotyping assays (TaqMan SNP Genotyping Assay by Applied Biosystems, Foster City, CA, USA). The assays were run on an ABI 7300 machine (Applied Biosystems, Foster City, CA, USA) and evaluated according to manufacturer's instructions. To ensure consistency between runs, samples of known genotypes were repeated in every analysis. For testing interactions with other associated genes, we used genotypes generated in previously published studies on this sample set [6,7].

### Statistical analysis

The Hardy-Weinberg equilibrium was checked by comparing observed to expected genotype frequencies in the control subjects, and tested using exact tests. Associations of particular SNPs with CD were evaluated using odds ratios (OR) with 95% confidence intervals (CI). Haplotype analysis was performed by estimating the haplotype frequencies by the expectation-maximization algorithm implemented in the R-project package 'haplo.stats' version 1.3.1. Association of haplotypes with the conditions was tested using log-linear modeling. Then, a potential interaction between the *CTLA4 *variants and other genetic factors associated with the autoimmune conditions were tested. The statistical analysis was performed using the R-project package 'SNPassoc' version 1.5-2 [24].

## Results

### Crude associations

The frequencies of the variants and respective OR are listed in Table 3. No crude associations with CD were found for the tested SNPs under the log-additive or dominant models. The genotype distributions in control subjects conformed to Hardy-Weinberg equilibrium in all SNPs (p > 0.20) except the rs1427676 (p = 0.014 in exact tests) which was therefore excluded from all further analyses.

**Table 3:** Distribution of genotypes of the studied *CTLA4 *polymorphisms^1^

Variants	Genotype frequency cases n = 333, controls n = 482			**Dominant model**^**2**^	**Log-additive model**^**2**^
**rs736611**	T/T	T/C	C/C	Genotype T/C + C/C	Allele C
CD	37%	48%	15%	63%	39%
Controls	34%	48%	18%	66%	42%
OR (95%CI)				0.89 (0.67 - 1.19)	0.86 (0.70 - 1.05)
					
**g.49A > G **(rs231775)	A/A	A/G	G/G	Genotype A/G + G/G	Allele G
CD	41%	46%	13%	59%	36%
Controls	40%	44%	16%	61%	38%
OR (95%CI)				0.85 (0.63 - 1.14)	0.89 (0.72 - 1.09)
					
**CT60 **(rs3087243)	G/G	G/A	A/A	Genotype G/A + A/A	Allele A
CD	33%	48%	19%	68%	43%
Controls	35%	48%	17%	65%	41%
OR (95%CI)				1.11 (0.82 - 1.50)	1.10 (0.90 - 1.35)
					
**JO31 **(rs11571302)	G/G	G/T	T/T	Genotype G/T + T/T	Allele T
CD	29%	50%	21%	71%	46%
Controls	31%	49%	20%	69%	44%
OR (95%CI)				1.12 (0.82 - 1.53)	1.09 (0.89 - 1.34)
					
**JO27-1 **(rs11571297)	A/A	A/G	G/G	Genotype A/G + G/G	Allele G
CD	29%	49%	22%	72%	47%
Controls	30%	50%	20%	70%	45%
OR (95%CI)				1.12 (0.82 - 1.53)	1.11 (0.91 - 1.36)

1: the rs1427676 polymorphism was excluded from the analyses as its distribution among healthy population did not conform to Hardy - Weinberg equilibrium.

2: Adjusted by gender

As the part of chromosome under the *CTLA4 *gene is divided into the several blocks [16] we performed a haplotype analysis using the five SNPs; no crude association with CD was observed (data not shown).

### *Interaction of the *CTLA4 *SNPs with variants in *IL23R *and *NOD2

**Figure 1:**
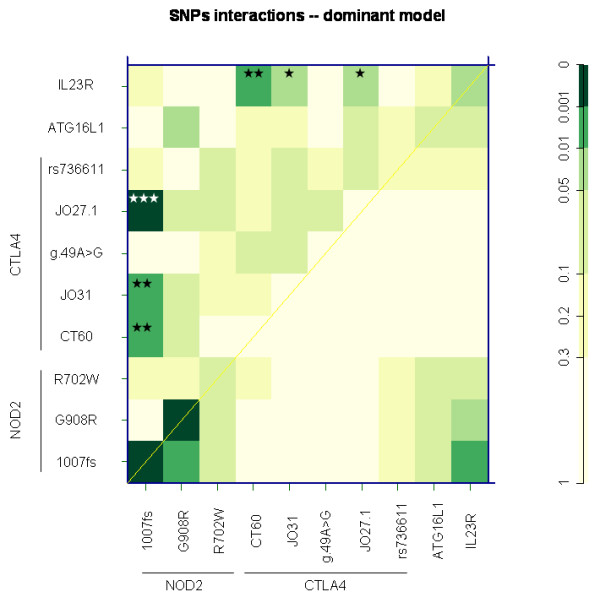
**SNPs interaction in the dominant model**. The upper triangle in matrix from this function contains the p values for the interaction (epistasis) log-likelihood ratio test (LRT). The diagonal contains the p values from LRT for the crude effect of each SNP. The lower triangle contains the p values from LRT comparing the two-SNP additive likelihood to the best of the single-SNP models [24]. P value of interactions: * P < 0.05; ** P < 0.01; *** P < 0.001.

We then tested possible interactions between variants in the *CTLA4 *and polymorphisms in other genes previously associated with CD: *NOD2 *gene p.Leu1007fsX1008 (c.3020insC), *IL23R *gene rs11209026 (c.1142G > A) [6,7], see Figure 1. This was done in dominant models using an R-project package 'SNPassoc' version 1.5-2 [24]. Significant interactions were observed between the three *CTLA4 *variants (CT60, JO31, JO27-1) and *NOD2 *p.Leu1007fsX1008, and the same variants in the *CTLA4 *and *IL23R *rs11209026.

For a quantification of the *CTLA4 *association stratified by the above *NOD2 *and *IL23R *polymorphisms see Table 4 a Table 5: the minor alleles of the CT60, JO31 and JO27-1 within the *CTLA4 *modified the risk of Crohn's disease in the stratum of subjects carrying the frameshift insertion p.Leu1007fsX1008 in *NOD2*, while no perceivable effect of *CTLA4 *was found in the stratum of p.Leu1007fsX1008 wild-type homozygotes. Similarly, the three *CTLA4 *variants clearly, albeit moderately, decreased the risk of CD in the stratum of subjects carrying minor alleles of rs11209026 within the *IL23R *(G/A and A/A), while no effect was observed in the *IL23R *wild-type homozygotes. The effect was observable also for the haplotype consisting of the three minor alleles of the variants in tight linkage disequilibrium, the CT60 "A", JO31 "T", JO27-1 "G" haplotype.

**Table 4:** Stratified analysis of the effect conferred by the *CTLA4 *CT60, JO31 and JO27-1 variants

*NOD2 *stratum **defined using the Leu1007fsX1008 polymorphism **^**1)**^	CT60 (rs3087243), A carriage rate (genotypes A/G, A/A)	JO31 (rs11571302), T carriage rate (genotypes G/T, T/T)	JO27-1 (rs11571297), G carriage rate (genotypes A/G, G/G)	**Haplotype "ATG" **^**4**^
***NOD2 *"+"**				
cases, n = 108	67 (62%)	69 (64%)	70 (65%)	
controls, n = 48	38 (79%)	40 (83%)	42 (88%)	
OR (95%CI) ^2)^	**0.43 **(0.19-0.95)	**0.35 **(0.15-0.83)	**0.26 **(0.1-0.68)	0.62 (0.37 - 1.05)
***NOD2 *"wt/wt"**				
cases, n = 224	157 (70%)	166 (74%)	168 (75%)	
controls, n = 434	277 (64%)	292 (67%)	294 (68%)	
OR (95%CI) ^2)^	1.33 (0.94-1.88)	1.37 (0.96-1.96)	1.42 (0.99-2.03)	1.21 (0.96. - 1.53)
**Heterogeneity between** ***NOD2*-defined strata **^3)^	**p = 0.011**	**p = 0.0042**	**p = 0.0011**	**p = 0.043**

Strata of the risk conferred by the p.Leu1007fsX1008 polymorphism of the *NOD2 *gene: the effect of *CTLA4 *is apparent in the stratum with an increased *NOD2*-associated risk

1) *NOD2 *"+": homozygous or heterozygous for the minor allele at the p.Leu1007fsX1008 polymorphism; *NOD2 *"wt/wt": wild-type homozygote at the p.Leu1007fsX1008 polymorphism. The *NOD2 *"+" category is associated with an increased risk of OR = 4.36, 95%CI 2.95 - 6.49 as compared to "wt/wt" category.

2) OR for the effect of the polymorphism in the specific stratum (*NOD2*"+" and *NOD2*"wt/wt"), adjusted for the effect of the *IL23R *p.381Gln variant and p.Gly908Arg, p.Arg702Trp in the *NOD2 *gene. Results significant at p < 0.05 are in bold.

3) Heterogeneity in the effect conferred by the CTLA4 polymorphisms was assessed between NOD2-defined strata using the Mantel-Haenszel test of homogeneity.

4) The implemented expectation-maximization algorithm did not allow individual imputation and counting of haplotypes.

**Table 5:** Stratified analysis of the effect conferred by the CTLA4 CT60, JO31 and JO27-1 variants

*IL23R *stratum **defined using the p.Arg381Gln polymorphism **^**1)**^	CT60 (rs3087243), A carriage rate (genotypes A/G, A/A)	JO31 (rs11571302), T carriage rate (genotypes G/T, T/T)	JO27-1 (rs11571297), G carriage rate (genotypes A/G, G/G)	**Haplotype "ATG" **^**4**^
***IL23R *"-"**				
cases, n = 21	10 (48%)	10 (48%)	10 (48%)	
controls, n = 50	42 (84%)	40 (80%)	40 (80%)	
OR (95%CI) ^2)^	**0.23 **(0.07-0.71)	**0.26 **(0.08-0.85)	**0.24 **(0.07-0.79)	**0.30 **(0.11 - 0.81)
***IL23R *"wt/wt"**				
cases, n = 312	214 (69%)	224 (72%)	227 (73%)	
controls, n = 432	276 (64%)	292 (68%)	296 (69%)	
OR (95%CI) ^2)^	1.26 (0.91-1.74)	1.22 (0.87-1.71)	1.21 (0.86-1.69)	1.20 (0.96 - 1.51)
**Heterogeneity between** ***IL23R*-defined strata **^3)^	**p = 0.0061**	**p = 0.011**	**p = 0.011**	**p = 0.030**

Strata of the risk conferred by the p.Arg381Gln polymorphism of the *IL23R *gene: the effect of *CTLA4 *is apparent in the stratum with an *IL23R*- associated protective effect

1) *IL23R *"-": homozygote or heterozygote for the *IL23R *p.381Gln allele; *IL23R *"wt/wt": wild-type homozygote at the p.Arg381Gln polymorphism. The *IL23R *"-" category is associated with a decreased risk of OR = 0.58, 95%CI 0.32 - 1.00 as compared to the "wt/wt" category.

2) OR for the effect of the polymorphism in the specific stratum (*IL23R *"-" or *IL23R *"wt/wt"), adjusted for the effect of p.Leu1007fsX1008, p.Gly908Arg, and p.Arg702Trp in the *NOD2 *gene. Results significant at p < 0.05 are in bold.

3) heterogeneity in the effect conferred by the *CTLA4 *polymorphisms was assessed between *IL23R*-defined strata using the Mantel-Haenszel test of homogeneity.

4) The implemented expectation-maximization algorithm did not allow individual imputation and counting of haplotypes.

### Genotype - phenotype analysis

Using a case-only design, we tested whether the phenotypic characteristics of the patients are dependent on carriage status of the minor alleles at *CTLA4 *variants.

Table 6 shows logistic regression with the outcomes of clinical characteristics and the three *CTLA4 *variants and the haplotype as the predictors. The pediatric-onset patients differed to the adult-onset patients in their frequencies of the minor allele at the CT60 (74% versus 63%, p = 0.03). The CT60, JO31 and JO27-1 SNPs, as well as their "ATG" haplotype retained their associations with the age at diagnosis after adjustment to the effect of the *NOD2 *variant (p.Leu1007fsX1008). No difference between pediatric and adult-onset group was found for the other two variants in the *CTLA4 *gene, data not shown.

**Table 6:** Genotype-phenotype analysis^1^

Outcome	CT60 (rs3087243) allele A	JO31 (rs11571302) allele T	JO27-1 (rs11571297) allele G	"ATG" haplotype
**Pediatric age at diagnosis**^**2**^	1.85 (1.12 - 3.03); **p = 0.014**	1.71 (1.03 - 2.85); **p = 0.035**	1.70 (1.02 - 2.84); **p = 0.039**	1.52 (1.09 - 2.11); **p = 0.014**
**Ileal involvement (L1)**	0.41 (0.24 - 0.70); **p = 0.0012**	0.45 (0.26 - 0.78); **p = 0.0052**	0.43 (0.24 - 0.74); **p = 0.0027**	0.70 (0.47 - 1.05); **p = 0.081**
**Ileocolonic involvement (L3) **^**3**^	1.97 (1.21 - 3.19); **p = 0.0059**	1.91 (1.16 - 3.13); **p = 0.010**	1.94 (1.18 - 3.20); **p = 0.0090**	1.54 (1.09 - 2.17); **p = 0.014**

1: OR with their 95%CI come from logistic regression analysis using dominant models, with the clinical phenotype as an outcome and *CTLA4 *CT60, JO31, JO27-1 minor variants and ATG haplotype as predictors; the models are adjusted for the p.Leu1007fsX1008 variant in the *NOD2 *gene.

2: Patients having been diagnosed before or at the age of 18 years.

3: Further, we tested interaction between *NOD2 *p.Leu1007fsX1008 variant and the *CTLA4 *ATG haplotype on development of L3. Comparing to wild haplotype on the background of the ATG haplotype the association of p.Leu1007fsX1008 *NOD2 *high risk variant was significantly weaker. P-value of the interaction between ATG haplotype and *NOD2 *p.Leu1007fsX1008 in the development of L3 was estimated 0.026.

The *CTLA4 *variants were weakly associated with the ileal-only (L1) and ileocolonic involvement (L3) in a dominant manner, while no association was observed with any of the remaining clinical characteristics: localization in the upper gastrointestinal tract, the stricturing or penetrating behavior of the disease, perianal disease, extraintestinal manifestation, or the need for abdominal surgery (data not shown).

## Discussion

The immunologic importance of the *CTLA4 *gene is in striking contrast to the lack of knowledge on the functional relevance of its numerous polymorphisms. Consequently, many groups have investigated various polymorphisms located within various regions of the gene. The first published study on *CTLA4 *variants in CD investigated the g.49A > G (rs231775) and g.-318C > T (rs5742909) in the Dutch and the Chinese populations, finding only an association with the age of onset [18]. Similarly, in a Hungarian work, no association of g.49A > G with CD was detected [21]. Since the work by Ueda et al [16] had been published on dissecting the association of *CTLA4 *with immunopathological diseases, further investigators focused on the CT60 polymorphism. This variant was studied in the Japanese [19] and the Spanish [20] populations, however no crude association with CD was detected. The G/G genotype of g.49A > G was associated with penetrating form of CD in the Japanese dataset [19]. No association within 2q33 chromosomal region has been found by genome-wide studies [4,5,8-12].

Thus, compelling evidence has been gathered against simple association of the disease itself with the polymorphisms of *CTLA4*. In line with these findings, we observed no crude association unless further genetic factors were taken into account. However, when *CTLA4 *was considered as a modifier of the effects conferred by the *NOD2 *and *IL23R *genes, possible interactions substantiated. Interactions in multifactorial immunopathological diseases are not infrequent: in CD, the interactions with the *NOD2 *gene were detected in the IBD5 locus [25], IBD6 locus [15], *TNFA *[26], *DLG5 *[27], *ATG16L1*[28], *IL23R *[29], *TLR4 *[30] and in *CD14 *[30]. The interaction was also found between IBD5 locus and *IL23R *[31] and between Toll-like receptor-9 polymorphisms and variants in *NOD2 *and *IL23R *[32].

The interactions we found for the CT60, JO31, JO27-1 variants of *CTLA4 *(or their haplotype) with the p.Leu1007fsX1008 variant of *NOD2 *may imply that the effect of the strongest risk variant within the *NOD2 *(p.Leu1007fsX1008) can be expressed better on the background of the common *CTLA4 *haplotype. This is suggestive of a complex pattern of gene-gene interaction that may merit pursuing further functional studies. Similarly, the risk haplotype of *CTLA4 *also interacts with the *IL23R *protective variants. This rather weak interaction can be also due to the relatively limited size of the dataset.

In addition to the modifying effect on the risk of the disease itself, we observed an association with the age at onset and the disease subphenotypes. Indeed, the impact of genetic factors in early-onset patients with CD seems to be stronger than in adult-onset patients (reviewed by de Ridder L et al. [33]). In our dataset, the age at diagnosis was associated with CT60, JO31 and JO27-1. Influence of *CTLA4 *variants on the age at diagnosis has been previously described by Xia et al, although with a different SNP (g.-318C > T) [18]. Moreover, their patients were divided into groups where 40 years of age was the cut off, not 18 years as in our study.

The *CTLA4 *was associated with the ileal and ileocolonic involvement in our case set: up to our knowledge, this is the first time when localization of CD is influenced by any variant within *CTLA4*. It should be however noted that these associations are weak, merit further investigation in other populations, and their clinical relevance can be only hardly envisaged. The ileal form of disease has been shown more common in adult-onset patients and more common in patients carrying minor variants of the *NOD2 *gene. A possible explanation of the association of *CTLA4 *with localization of disease might be found in the interaction between *CTLA4 *and *NOD2 *gene.

Similarly to Machida et al [19] we also tested whether the g.49A > G variant influences the occurrence of penetrating disease, but we were not able to confirm this association. However, the genetic background between Japanese and Czech populations differs markedly.

## Conclusions

We present a study of genetic association of polymorphisms within the *CTLA4 *gene with CD and its subphenotypes, using a representative set of markers previously reported from other studies. We observed interactions of the *CTLA4 *haplotype with variants in *NOD2 *and *IL23R *genes, and detected an effect of three variants of the *CTLA4 *on the age at diagnosis and localization of the disease.

## Competing interests

The authors declare that they have no competing interests.

## Authors' contributions

OH, PD and MLe performed the experiments; JB, JN, LV, MLu coordinated and performed the collection of the samples and were also involved in editing the manuscript; OC and OH designed the study and wrote the manuscript. All authors read and approved the final manuscript.

## Pre-publication history

The pre-publication history for this paper can be accessed here:

http://www.biomedcentral.com/1471-2350/11/91/prepub
